# Reinforcement Learning Approach to Optimizing Profilometric Sensor Trajectories for Surface Inspection

**DOI:** 10.3390/s25072271

**Published:** 2025-04-03

**Authors:** Sara Roos-Hoefgeest, Mario Roos-Hoefgeest, Ignacio Álvarez, Rafael C. González

**Affiliations:** 1 Department of Electrical, Computer Electronics and Systems Engineering, University of Oviedo, 33003 Oviedo, Spain; ialvarez@isa.uniovi.es (I.Á.); rcgonzalez@uniovi.es (R.C.G.); 2 CIN Advanced Systems Group, 33211 Gijón, Spain; marioroos2502@gmail.com

**Keywords:** industrial robots, trajectory planning, reinforcement learning, automatic optical inspection, surface profile, laser radiation, NDT

## Abstract

High-precision surface defect detection in manufacturing often relies on laser triangulation profilometric sensors for detailed surface measurements, providing detailed and accurate surface measurements over a line. Accurate motion between the sensor and workpiece, usually managed by robotic systems, is critical for maintaining optimal distance and orientation. This paper introduces a novel Reinforcement Learning (RL) approach to optimize inspection trajectories for profilometric sensors based on the boustrophedon scanning method. The RL model dynamically adjusts sensor position and tilt to ensure consistent profile distribution and high-quality scanning. We use a simulated environment replicating real-world conditions, including sensor noise and surface irregularities, to plan trajectories offline using CAD models. Key contributions include designing a state space, action space, and reward function tailored for profilometric sensor inspection. The Proximal Policy Optimization (PPO) algorithm trains the RL agent to optimize these trajectories effectively. Validation involves testing the model on various parts in simulation and performing real-world inspection with a UR3e robotic arm, demonstrating the approach’s practicality and effectiveness.

## 1. Introduction

Surface inspection is a critical aspect of quality control in many industries, ensuring that manufactured components meet strict standards and function reliably. Traditional inspection methods often rely on manual processes performed by human operators, which struggle to detect micron-scale defects and meet the accuracy and efficiency demanded by modern manufacturing. As industries strive for higher precision, automated inspection technologies based on high-precision sensors have become critical [1]. Among them, laser triangulation has gained prominence due to its superior combination of precision and efficiency [2,3], enabling detailed surface topography analysis for defect detection [4,5]. Nevertheless, effective inspection of a whole part requires coordinated sensor motion, highlighting the need for optimized robotic trajectory planning.

Robotic arms are commonly used to ensure complete coverage during surface inspection, systematically scanning surfaces to acquire detailed defect information [6,7]. However, the integration of robotics with profilometric sensors for surface inspection remains an underexplored area. Chen et al. [8] address this gap by proposing a method for defect detection using a 6-degree-of-freedom manipulator equipped with a line scanner and depth sensor, optimizing both local inspection trajectories and global scanning efficiency. Similarly, Li et al. [9] present a trajectory planning approach that segments the surface into regions and optimizes scanning paths using a minimum enclosing rectangle.

Traditional robotic trajectory planning methods often rely on rule-based or optimization approaches, but these can struggle with complex surface geometries and require extensive manual tuning. Recently, Reinforcement Learning (RL) has emerged as a promising alternative, offering adaptability and real-time optimization. While RL has been widely explored in various robotics applications [10,11,12,13], its potential for trajectory planning in industrial inspection tasks remains largely untapped. Han et al. [14] provide a comprehensive review of different applications of deep RL in robotic manipulators, highlighting key challenges such as discrepancies between simulation models and real-world dynamics. One major issue, known as the sim-to-real problem, occurs when RL models trained in simulation fail to generalize well to real-world environments due to differences in sensor data accuracy, lighting conditions, or object textures.

To mitigate these challenges, we leverage a realistic simulator introduced in our previous work [15], which accurately replicates profilometric laser triangulation sensor measurements. Additionally, trajectory generation in robotics is inherently multidimensional, complicating the learning and optimization process. Ji et al. [16] emphasize that most RL-based research in robotics focuses on mobile robot navigation due to its well-developed theoretical foundation and simpler implementation [17].

Surface inspection with profilometric sensors is typically conducted in a straight line. For larger workpieces requiring multiple scans, parallel passes often follow boustrophedon-like trajectories [18]. Building on these conventional approaches, we propose an RL-based method to optimize sensor movements dynamically, ensuring complete coverage while minimizing trajectory complexity. Each scan advances in a predetermined direction while the sensor adjusts its height and pitch angle over the surface, keeping other orientations constant. We focus on the sensor’s advance speed, height adjustment, and pitch orientation to simplify the trajectory optimization process while maintaining high accuracy and reducing the dimensionality of the action space.

Reinforcement learning techniques offer effective solutions for problems with low dimensional action spaces. Our proposal takes advantage of them for optimizing the generation of surface inspection trajectories. Unlike traditional optimization techniques that rely on predefined heuristics, RL continuously refines control policies based on learned experiences, improving adaptability to diverse inspection scenarios. Despite significant advancements in RL algorithms, their application in industrial inspection remains relatively underexplored compared to other robotics fields. This gap highlights the necessity for further research in this area.

Most studies in the field of inspection applications focus on the View Planning Problem (VPP). The aim is to determine the optimal poses for capturing images or making measurements using 3D vision sensors. For example, Jing et al. [19] combined VPP and Coverage Path Planning (CPP) to automate robotic trajectory generation for surface inspection, using Monte Carlo algorithms and greedy search techniques to optimize scanning efficiency. Similarly, Landgraf et al. [20] presented an automatic viewpoint planning method for 3D surface inspection, employing RL algorithms such as Q-learning, PPO, and DQN to determine optimal viewpoints and guide robotic movements.

However, traditional VPP approaches are insufficient for laser triangulation profilometric sensors, which require precise measurements along predefined paths rather than broad 3D views. A tailored path-planning method is needed to account for these sensors’ unique linear scanning nature. To bridge this gap, we introduce an RL-based method for generating optimized inspection trajectories specifically for profilometric sensors.

We apply the Proximal Policy Optimization (PPO) algorithm, as proposed by OpenAI [21], to optimize inspection trajectories. PPO balances ease of implementation, sampling efficiency, and hyperparameter tuning simplicity, offering competitive performance compared to other advanced RL algorithms. Unlike alternative approaches such as SAC and TD3, which often require extensive fine-tuning, PPO provides a robust training process with fewer hyperparameter adjustments, making it more reliable for constrained robotic tasks. PPO has proven effective in applications such as view planning for inspections [20] and pick-and-place tasks involving 7-degree-of-freedom robotic arms [22,23].

In our proposed approach, we train a PPO model offline using a simulated scenario designed to replicate real-world inspection conditions. The state space, action space, and reward function are specifically designed to guide the system in generating precise scanning trajectories. The model dynamically adjusts the sensor’s position and orientation along the scanning path, maintaining optimal distance and alignment with the object to improve data quality and reduce noise. To ensure robustness, the training environment includes surfaces with varying heights, orientations, and curvatures, providing sufficient variability for effective learning. Once trained, the model is applied to generate efficient scanning paths for real objects without requiring adaptation to specific object geometries.

Experimental results across various objects validate the effectiveness and adaptability of our approach, demonstrating its practical application in real-world inspection scenarios. By leveraging RL to optimize surface inspection trajectories, our work advances the integration of profilometric sensors with robotic automation, addressing a significant gap in the literature.

## 2. Materials and Methods

**Figure 1:**
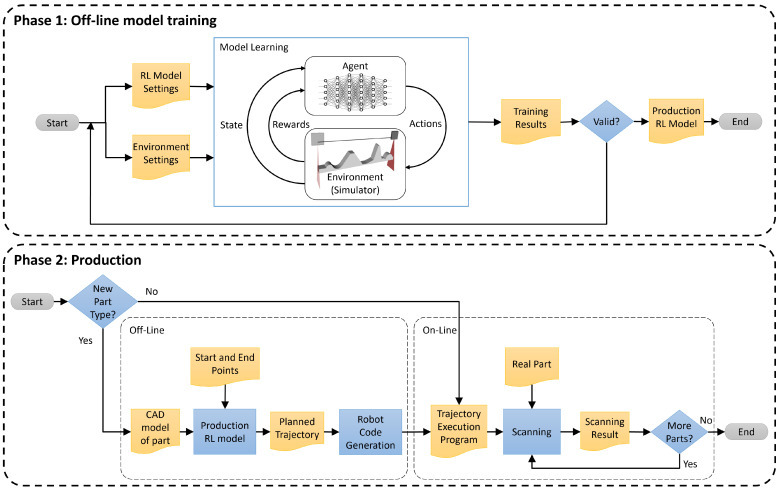
Schematic of the proposed method. In a first phase, a reinforcement learning model is trained offline using a simulated environment. Once a suitable model is achieved, it can be applied in the production phase. This second phase is divided in two steps. Using the CAD model of the part to be inspected and the trained model, an optimal inspection trajectory is computed offline, and a program to follow that trajectory is automatically generated. The second step consists of executing the generated program to inspect every part in a production batch.

In this section, we present the proposed approach for generating inspection trajectories using profilometric sensors and reinforcement learning techniques. The goal is to improve the inspection trajectories for a workpiece, which are typically scanned following a straight line between two points or, in the case of larger pieces, through a boustrophedon path. Each pass along the scanning path is planned to keep the sensor at its optimal orientation and distance from the part at every point. This involves dynamically adjusting the position and tilt (pitch) of the sensor to ensure a consistent pose between the sensor and the surface at all times. The other two sensor orientations will be fixed, allowing for accurate and uniform data capture. In addition, the profile spacing will be taken into account to ensure full scanning coverage. Figure 1 shows the schematic of the proposed method.

To train the RL algorithms, we used a simulated environment replicating the conditions of the real system described in our previous work [15]. This simulator emulates the measurements of a laser triangulation profilometric sensor, including sensor and speckle noise generated by the object’s surface. Thus, a realistic and controlled training environment is obtained.

The RL model is initially trained with a generic part, and once the model is trained, it can be applied to different pieces with varying geometries, without needing retraining. This enables the generation of inspection trajectories that can be used across a wide range of parts, demonstrating the flexibility and robustness of the approach for real-world applications.

The state space is constructed using the position and orientation of the robot’s end-effector. This allows for generalization of the approach and facilitates the transfer of the method to different robotic configurations. Additionally, the state also includes other parameters, such as the mean profile distance, the incidence angle, and the spacing between consecutive scans.

The action space is defined by relative increments in the sensor’s position and tilt angle, allowing for precise adjustments and smooth movements of the sensor. The reward function consists of three key components: the distance between the sensor and the surface, the alignment of the sensor with the surface normal, and the spacing between consecutive scans. This comprehensive reward function encourages optimal behaviors in terms of distance, orientation, and sensor advancement.

Next, each component is described in detail, providing a thorough understanding of its design and implementation.

### 2.1. Reinforcement Learning (RL): Basic Concepts

**Figure 2:**
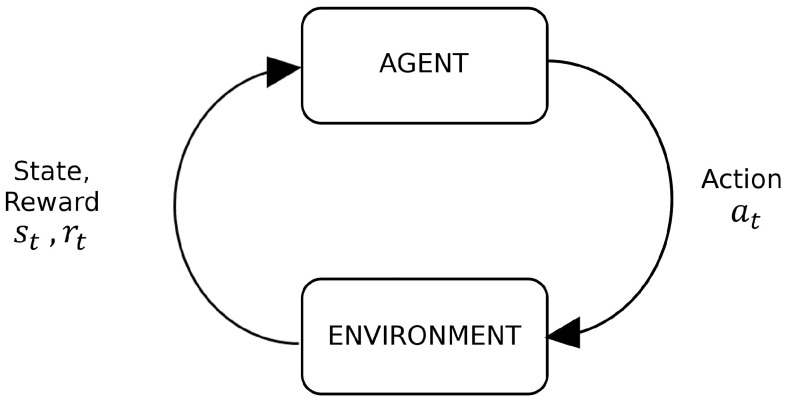
Basic scheme of a reinforcement learning algorithm.

Reinforcement Learning (RL) is a machine learning paradigm inspired by behavioral psychology, aimed at optimizing decision-making by agents to maximize cumulative rewards over time [24]. In RL, an agent interacts with an environment by observing its state, taking actions, and receiving rewards based on those actions. The agent’s goal is to maximize the total reward accumulated, which involves continuously adapting its strategy to improve performance. Figure 2 illustrates the basic structure of a reinforcement learning algorithm.

The agent operates within a defined environment, where actions are selected from an action space (A) and influence the state of the environment. The state space (S) encompasses all possible states of the environment. Rewards are given for actions taken, and the objective is to accumulate the highest possible reward over time.

The problem is modeled as a Markov Decision Process (MDP), defined by states, actions, rewards, transition probabilities, and the initial state distribution. The agent follows a policy (π), which can be deterministic or stochastic, guiding its decision-making process.

The core aim of RL is to find the optimal policy ($\pi^{*}$) that maximizes the expected return, given as follows:

(1) $$J\left(\pi \right)=E_{\pi }\left[r\left(s_{t},a_{t}\right)\right]$$

The optimization problem is expressed as follows:

(2) $$\pi^{*}=\arg\max_{\pi }J\left(\pi \right)$$

### 2.2. Scanning Characteristics for Surface Inspection with Profilometric Sensors

The quality of the data captured by the laser triangulation profilometric sensors is significantly influenced by factors related to the sensor’s position relative to the inspected surface [9]. Proper consideration of these parameters during trajectory planning is crucial for achieving accurate and comprehensive surface inspection.

One of these crucial parameters is the optimal working distance ($W_{d}$), which denotes the ideal distance between the sensor and the object’s surface. This distance ensures optimal precision of the captured data by positioning the laser source at the scanning reference plane, typically located at the midpoint of the depth of field.

**Figure 3:**
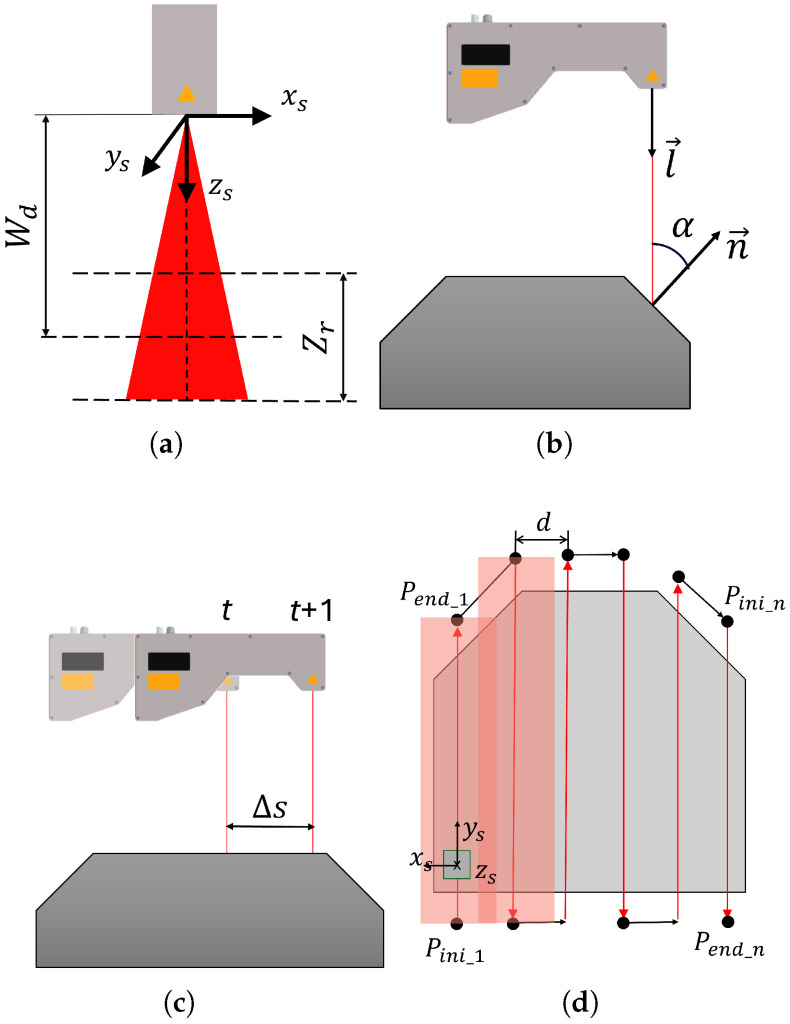
Main parameters of a profilometric sensor. (**a**) $W_{d}$ and $Z_{r}$. (**b**) Direction angle α. (**c**) Distance between two consecutive profiles Δs. (**d**) Boustrophedon trajectory (zenital view): Multiple parallel passes are made, separated by distance *d*, with overlapping areas between passes. Gray square represents the sensor; red lines show data capture paths, and black lines indicate intermediate movements between passes.

The depth of field ($Z_{r}$) refers to the range of distances within which the sensor can capture surface data during a single scan (Figure 3a). Assuming a point in the scanner’s coordinate system is $\left(x_{s},0,z_{s}\right)$, the Equation (3) must be satisfied. Operating within this range is critical, as it minimizes noise levels associated with deviations from the optimal working distance. Studies [25] have demonstrated that maintaining proximity to the optimal working distance reduces noise, thereby enhancing measurement accuracy.

(3) $$W_{d}-\frac{Z_{r}}{2}\leq z_{s}\leq W_{d}+\frac{Z_{r}}{2}$$

Another crucial parameter is the direction angle (α), which signifies the angle between the sensor’s orientation vector $\vec{l}$ and the normal vector $\vec{n}$ of the workpiece surface (Figure 3b. This angle is computed using Equation (4). As the direction angle increases, there is a higher likelihood of introducing noise into the capture. This phenomenon occurs because the scanner may capture unwanted reflections of the laser light and variations in surface reflectivity, negatively impacting the data quality. Previous studies [25,26] have empirically shown how noise levels correlate with the direction angle, highlighting its significance in achieving precise surface capture.

(4) $$\alpha =acos\left(-\vec{l}\;\cdot \;\vec{n}\right)$$

Additionally, the distance between profiles (Δs) determines the density of points between consecutive scan profiles. Adequate point density ensures comprehensive coverage and accuracy of the inspected surface, particularly in areas with small features or irregular surfaces where a lower density might compromise inspection quality. See Figure 3c.

In addition to considering these parameters, it is crucial to choose the appropriate type of trajectory to achieve a complete scan of the surface of the piece. In laser profilometer inspection applications, one of the most common strategies is to use boustrophedon paths [18,27]. In a boustrophedon scan, the sensor moves in a straight line along one axis until it reaches the edge of the surface to be inspected. Then, it shifts a predetermined distance laterally and changes direction to move in the opposite direction along the initial axis. This pattern of movements is repeated until the entire surface is covered. In the context of surface inspection, this method is highly efficient in ensuring that every area of the piece’s surface is scanned without omissions, thereby maximizing coverage and inspection accuracy.

Considering these types of trajectories, the profilometric sensor collects data only during the passes along the surface of the piece. In Figure 3d, these trajectories are shown in red, from the initial point of a pass ($P_{ini_{i}}$) to its end point ($P_{fin_{i}}$), where *i* denotes the number of parallel passes. The movement of the robot between each pass is shown in black. The distance between passes, *d*, is carefully adjusted to ensure that the scans overlap adequately, thereby completely covering the piece.

### 2.3. Simulated Environment

**Figure 4:**
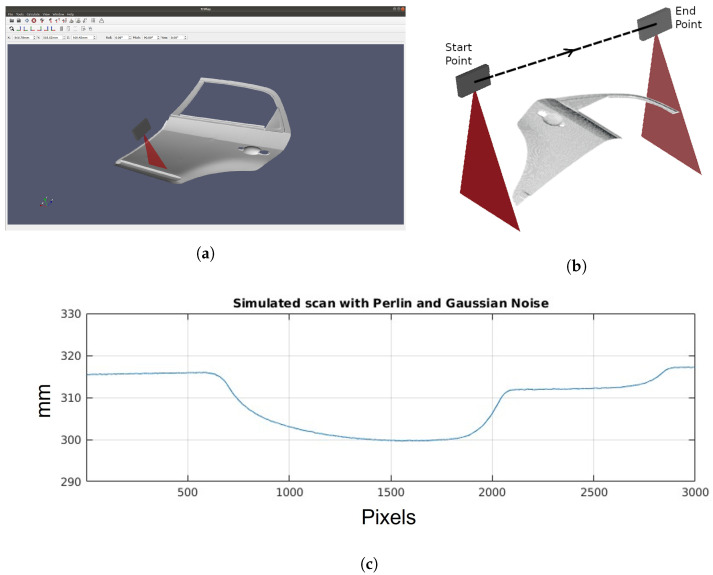
(**a**) View of the simulated environment with the profilometric sensor and a part to be inspected. (**b**) 3D point cloud result during the scan of the CAD model section. Start and end points of the trajectory can be seen. (**c**) Profile obtained during the scan simulation.

To effectively train reinforcement learning algorithms, we use our previously developed simulator, see Figure 4a. This simulator replicates the behavior of a high-resolution laser triangulation profilometric sensor by combining a geometrical model with noise simulations. The inspected part’s geometry is represented using an STL CAD model, and the sensor’s movement relative to the surface is simulated to generate depth measurements. The simulator incorporates Gaussian noise, representing the sensor’s depth resolution, and Perlin noise, simulating speckle noise due to material roughness. By integrating these noise components, the simulator allows for precise measurement reproduction, minimizing the sim-to-real gap. For further details on configuring and using the simulator, please refer to the original paper [15].

In each iteration of the simulator, several critical parameters are obtained that will be used later by the RL algorithm. First, the distance profile is captured, a fundamental representation provided by any profilometric sensor. Additionally, the 3D position of the scanned points of the CAD model is collected, providing detailed information about the surface geometry of the object. Furthermore, the simulator also provides the normal vectors at those points on the object’s surface. Figure 4b shows the resulting 3D point cloud obtained from the scan, highlighting the start and end points of the trajectory, and Figure 4c a profile generated during the simulation.

### 2.4. State Space

As previously mentioned, the position and orientation of the end-effector are used instead of relying on the positions and velocities of the robot’s joints. This choice simplifies the state space and facilitates the transfer of the method to different robotic configurations without the need for specific adjustments in the joints. Mathematically, the state *S* is defined as a tuple as follows:

(5) S={P(x,y,z),θ,D,α,Δs}

**Figure 5:**
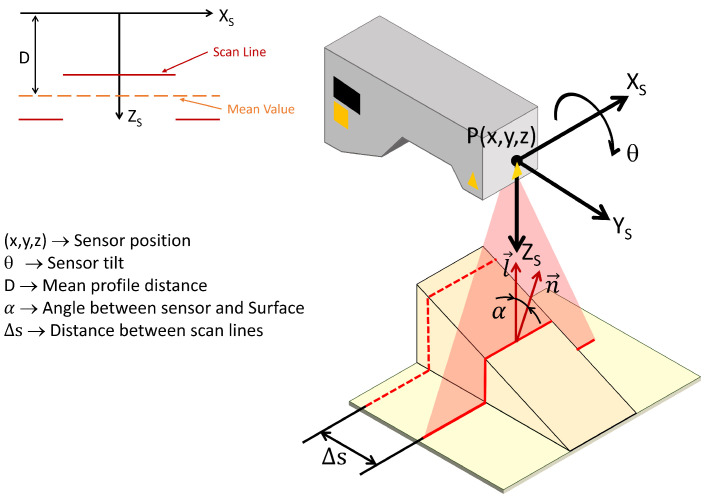
The configuration of the scanning system representing the state space variables. P(x,y,z) represents the position of the sensor, θ represents sensor orientation, *D* represents the average sensor distance, α is the angle between the sensor orientation $\left(\vec{l}\right)$ and the surface normal $\left(\vec{n}\right)$, and Δs refers to the distance between consecutive scan lines.

Here, P(x,y,z) represents the position of the end-effector, while θ denotes its tilt. The parameters *D*, α, and Δs correspond to the mean profile distance obtained from the scan, the angle between the sensor and the surface, and the advance between consecutive scans in the 3D space, respectively. Figure 5 shows a schematic representation of the selected state space variables.

To ensure training stability and improve the model’s generalization, each state-space variable is normalized individually using min–max scaling. The position of the end-effector P(x,y,z) is scaled according to the robot’s workspace limits, ensuring consistency across different spatial configurations. The tilt angle θ is normalized based on mechanical constraints to reflect realistic orientation bounds. The average sensor-to-surface distance *D* is normalized according to the sensor’s specified measurement range, providing accurate scaling relative to operational conditions. Similarly, the angle α is scaled within empirically determined limits, ensuring proper sensor alignment. Finally, the advancement between consecutive scans Δs is normalized according to the experimentally validated movement increments, guaranteeing smooth and controlled scanning operations. This normalization procedure ensures balanced contributions from each parameter during the training process, reducing the potential for instability or numerical biases.

### 2.5. Action Space

The action space is defined by the increments in the position and tilt angle of the inspection sensor. These increments are defined relative to the sensor’s own coordinate system. Mathematically, the action space is represented by Equation (6).

**Figure 6:**
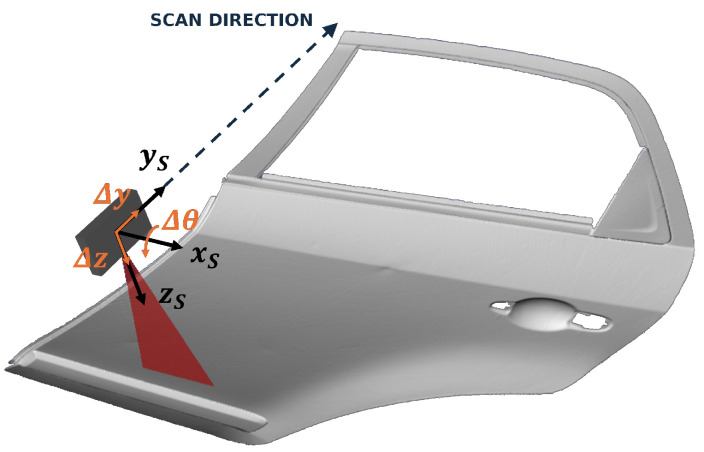
The simulation environment, representing the action space as unit vectors (in orange). Δy refers to the increment in position in the scanning direction, Δz refers to the increment in the vertical direction (Z), and Δθ denotes the change in the sensor’s pitch orientation.

(6) A={Δy,Δz,Δθ}

where Δy refers to the increment in position in the sensor’s forward direction (Y), which will be previously defined by a unit vector indicating the scanning direction. Δz refers to the increment in position in the sensor’s vertical direction (Z), controlling the height of the end-effector relative to the part. Δθ denotes the change in the sensor’s pitch orientation, which is the rotation around the X-axis. This is represented in Figure 6.

The action space is defined as continuous, meaning that actions span a continuous range of values rather than discrete ones. This approach ensures smooth and controlled sensor movements to avoid abrupt changes that could affect measurement accuracy or cause collisions with the workpiece. Equation (7) establishes the limits for each type of action in the continuous space. Here, Δy, Δz, and Δθ are constrained to values between $\pm \Delta y_{\max}$ millimeters, $\pm \Delta z_{\max}$ millimeters, and $\pm \Delta \theta_{\max}$ degrees, respectively.

(7) $$\begin{array}{c} \Delta y\in [-\Delta y_{\max},\Delta y_{\max}]\\ \Delta z\in [-\Delta z_{\max},\Delta z_{\max}]\\ \Delta \theta \in [-\Delta \theta_{\max},\Delta \theta_{\max}] \end{array}$$

To ensure smooth and safe sensor movement, actions are dynamically adjusted based on environmental observations. When the sensor is farther from the part surface than the optimal working distance $W_{d}$, limits are applied to the sensor’s displacement in the Z direction Δz to bring it closer to the surface in a controlled manner. Conversely, if the sensor is too close, displacements in the negative direction are limited, as per Equation (8).

(8) $$\Delta z=\left\{\begin{array}{cc} \mathrm{clip}(\Delta z,0,\Delta z_{\max}) & \mathrm{if}\;(D-W_{d})\geq 0\\ \mathrm{clip}(\Delta z,-\Delta z_{\max},0) & \mathrm{if}\;(D-W_{d})<0 \end{array}\right.$$

Here, clip(x,a,b) limits the value of *x* to between *a* and *b*, ensuring that the actions are within the permitted range according to Equation (9).

(9) $$\mathrm{clip}\left(x,a,b\right)=\left\{\begin{array}{c} a\;\mathrm{if}\;x\leq a\\ b\;\mathrm{if}\;x\geq b\\ x\;\mathrm{else} \end{array}\right.$$

Similarly, if the sensor’s direction angle (α) with respect to the surface normal is positive, indicating excessive tilt, limits are applied to the angular displacement Δθ to correct the sensor’s orientation. Conversely, if the tilt angle is negative, limits are applied to the angular displacement in the opposite direction to keep the sensor properly aligned with the inspected surface. This is represented in Equation (10).

(10) $$\Delta \theta =\left\{\begin{array}{cc} \mathrm{clip}(\Delta \theta ,0,\Delta \theta_{\max}) & \mathrm{if}\;\alpha \geq 0\\ \mathrm{clip}(\Delta \theta ,-\Delta \theta_{\max},0) & \mathrm{if}\;\alpha <0 \end{array}\right.$$

### 2.6. Reward Function

In reinforcement learning, an effective reward model is essential for guiding the agent’s behavior. Our proposed reward function R(s,a) consists of three distinct components, each capturing different aspects of the inspection process. Mathematically, this function is expressed as shown in Equation (11).

(11) $$R\left(s,a\right)=w_{d}R_{D}+w_{\alpha }R_{\alpha }+w_{\Delta s}R_{\Delta s}$$

$R_{D}$ represents the reward related to the distance between the sensor and the inspected surface, $R_{\alpha }$ denotes the reward related to the alignment of the sensor’s orientation with the normal of the inspected object’s surface, and $R_{\Delta s}$ captures the reward associated with the sensor’s movement between consecutive scans in the 3D space corresponding to the point cloud of the inspected piece. $w_{d},w_{\alpha },w_{\Delta s}$ represent the weights that each component contributes to the overall reward function.

The proposed rewards are in the range [0,−1], as the reward function aims to incentivize the agent to perform actions that improve the inspection process. The maximum value of 0 is assigned when the optimal goal is reached, while negative values indicate penalties for deviations from the desired behavior.

#### 2.6.1. Distance Reward $\left(R_{D}\right)$

To ensure that the sensor maintains an optimal distance from the inspected surface, a distance reward function $R_{D}$ is defined as a continuous penalty function that decreases as the absolute difference between the observed distance and the optimal working distance $W_{d}$ increases. The reward function is formulated as follows:

(12) $$R_{D}=-\frac{{\left(W_{d}-D\right)}^{2}}{{\left(\frac{Z_{r}}{2}\right)}^{2}}$$

where $W_{d}$ represents the optimal working distance, *D* the observed distance during scanning, and $Z_{r}$ the specified working range of the sensor. This results in a parabolic function with values between [−1,0], corresponding to 0 when operating at the optimal working distance and −1 at the sensor’s range limits. If the distance is outside this range, the penalty is maximum (−1).

The quadratic penalty function was chosen because it provides a smooth and symmetric decrease in reward as the sensor deviates from the optimal working distance $W_{d}$. This formulation ensures a more significant penalty for larger deviations while maintaining a gradual variation that facilitates stable training. Unlike a linear penalty, which does not sufficiently differentiate between small and large errors, the quadratic form better reflects the non-linear nature of measurement accuracy loss with distance.

#### 2.6.2. Orientation Reward $\left(R_{\alpha }\right)$

To make the agent align its orientation with the surface normal, we introduce an orientation reward model ($R_{\alpha }$). This model is designed to minimize the angular disparity between the sensor direction and the surface normal vector. The function is defined as a continuous penalty function that approaches 0 as the absolute orientation difference decreases.

(13) $$R_{\alpha }=\max\left(-1,-\frac{\alpha^{2}}{\alpha_{\max}^{2}}\right)$$

where α is the angular difference between the sensor’s orientation and the surface normal, and $\alpha_{\max}$ is the maximum allowed angular disparity threshold. This model encourages the agent to maintain close alignment with the surface normal, optimizing the quality of the inspection.

#### 2.6.3. Movement Reward $\left(R_{\Delta s}\right)$

In addition to optimizing the distance and orientation of the sensor, ensuring smooth forward movement is crucial for comprehensive surface coverage. Forward scanning movement ensures that each scanned 3D profile extends beyond the previous one, facilitating thorough inspection. The reward function $R_{\Delta s}$ is expressed as follows:

(14) $$R_{\Delta s}=\max\left(-1,-\frac{{\left(\Delta s-\Delta s_{opt}\right)}^{2}}{\Delta s_{opt}^{2}}\right)$$

This function penalizes the agent when the scanning spacing Δs is negative, indicating backward movement within the inspection area. Likewise, it behaves parabolically with respect to the scanning spacing Δs. When the spacing is equal to twice the optimal value $\Delta s_{opt}$, the reward reaches its minimum value of −1. This indicates a strong penalty for excessively large spacings. As the spacing decreases from this point, the reward gradually increases, reaching a maximum value of 0 when the spacing is exactly equal to the optimal value. Therefore, the reward function motivates the agent to maintain spacing close to the optimal, as both above and below-optimal values result in a decrease in reward.

### 2.7. RL Algorithm: Proximal Policy Optimization

The Proximal Policy Optimization (PPO) algorithm [21] is an advanced policy gradient method designed to enhance the efficiency and stability of reinforcement learning compared to older algorithms like Advantage Actor–Critic (A2C) and Deterministic Policy Gradient (DPG). PPO improves upon Trust Region Policy Optimization (TRPO) by using first-order gradients and a clipped objective function to simplify and accelerate training.

PPO’s key feature is its clipped surrogate loss function, which limits policy updates to prevent excessive changes and ensure stable training. The objective function is defined as follows:

(15) $$J_{\mathrm{clip}}\left(\pi \right)=E\left[\min\left(r\left(\pi \right)\hat{A},\mathrm{clip}\left(r(\pi ),1-\epsilon ,1+\epsilon \right)\hat{A}\right)\right]$$

Here, r(π) is the ratio of probabilities under the new and old policies, $\hat{A}$ is the estimated advantage, and ϵ is a hyperparameter controlling the extent of policy change. The ratio r(π) is calculated as follows:

(16) $$r\left(\pi \right)=\frac{\pi_{\mathrm{new}}\left(a_{t}|s_{t}\right)}{\pi_{\mathrm{old}}\left(a_{t}|s_{t}\right)}$$

For the PPO algorithm, key hyperparameters include the neural network architecture, which specifies the number of hidden layers and units per layer. ReLU is typically used as the activation function to introduce non-linearity. The learning rate controls the size of weight updates, affecting learning speed and stability. The clip ratio restricts policy changes between updates to maintain training stability, while the epoch parameter defines the number of times the dataset is processed during training.

## 3. Results

This section presents the experiments conducted to validate and evaluate the proposed reinforcement learning (RL) methods for generating inspection trajectories. This study compared RL-optimized scanning paths with traditional approaches, such as straight-line and boustrophedon patterns, to assess the RL method’s ability to maintain optimal sensor distance and orientation. Initial tests were conducted in simulation, followed by real-world experiments to validate the transferability of the RL-generated solutions to practical scenarios.

These algorithms were implemented using the open-source library stable-baselines3 [28], which provides enhanced implementations of reinforcement learning algorithms based on OpenAI. To analyze and process the obtained results, we used MATLAB 2023b.RoboDK v5.7.0 [29] is used to translate the trajectory points into precise joint movements for implementation in real-world experiments.

**Figure 7:**
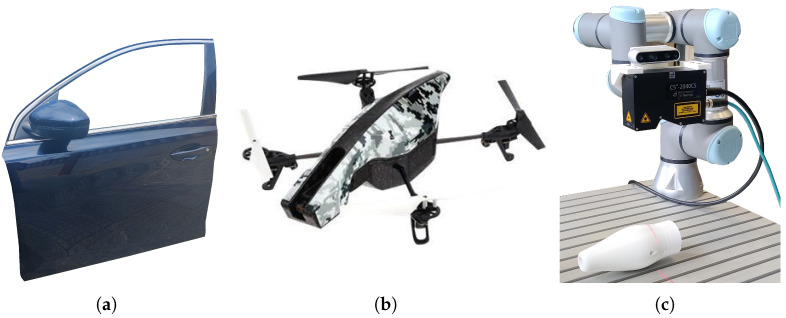
Parts used in experiments. (**a**) Car door. (**b**) Parrot Drone. (**c**) Pen holder and experimental setup: The UR3e robotic arm from Universal Robots, equipped with an AT sensor for scanning.

Three different objects were used in the tests: a car door, a drone body, and a pen holder (see Figure 7). Each object was carefully selected to encompass a diverse range of geometries, materials, and inspection challenges, ensuring a comprehensive evaluation of our approach.

The car door, made of steel, is a predominantly flat structure with smooth curvature transitions. This type of surface is common in industrial inspection and requires broad, uniform scanning coverage. The drone body, composed of plastic, features complex contours, tight areas, and multiple angular surfaces, posing challenges in maintaining optimal sensor positioning and ensuring full coverage of intricate details. Lastly, the pen holder, a 3D-printed part made of PLA (polylactic acid), is a compact object with continuous curvature variations, testing the system’s ability to adapt to small-scale structures and smoothly transition between different surface inclinations.

By incorporating objects of different sizes, shapes, and materials, this experimental setup effectively validates the versatility and robustness of our method across a wide spectrum of real-world inspection scenarios.

The scanning system is composed of a 6 DOF UR3e robotic arm [30] equipped with a triangulation laser profilometer model AT-C5-2040-CS-14-100 (Universal Robots USA, Inc., Novi, MI, USA). The complete configuration of the inspection system, which includes the UR3e robotic arm equipped with the profilometric sensor, can be seen in Figure 7c. The main parameters of the sensor are obtained from its datasheet [31] and detailed in Table 1.

### 3.1. Training Process

The training process of the RL model for trajectory optimization in robotic inspection was developed using a detailed simulation environment, the characteristics of which are explained in [15]. In this context, a profilometric sensor was simulated with the same specifications as the Automation Technology model AT-C5-2040-CS-14-100, whose main parameters are detailed in Table 1. It is important to note that this setup is designed to generalize based on input parameters, allowing for adjustments to different working distances, for example.

**Figure 8:**
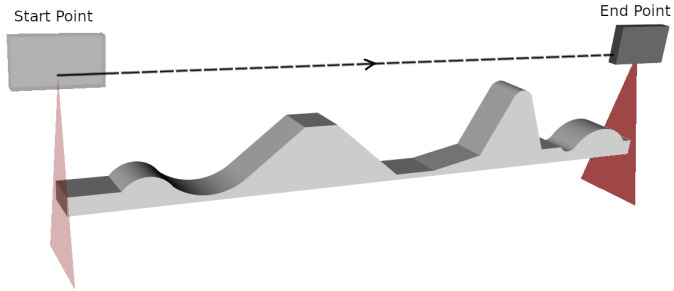
Environment used for RL training. The CAD model of the training piece is shown, along with the start and end poses of the trajectory to be optimized. The piece was designed to simulate a variety of real-world inspection conditions, including orientation changes, height variations, and flat surfaces.

The training piece was designed to simulate the diverse conditions found in real inspection scenarios, featuring orientation changes, height variations, and flat surfaces. This 1050 mm × 150 mm × 50 mm piece, shown in Figure 8, was modeled using 3D software. Each training episode corresponds to a start and end point based on the scanning direction and piece dimensions.

In the experiments, the action space is continuous, meaning that actions are expressed as values within a continuous range rather than discrete values. Specifically, these actions are limited within the interval of [−1, 1], where position increments are measured in millimeters and pitch angles in degrees. Table 2 outlines the key hyperparameters for the PPO algorithm. These parameters were selected based on default values recommended by the authors of the stable-baselines3 library.

**Figure 9:**
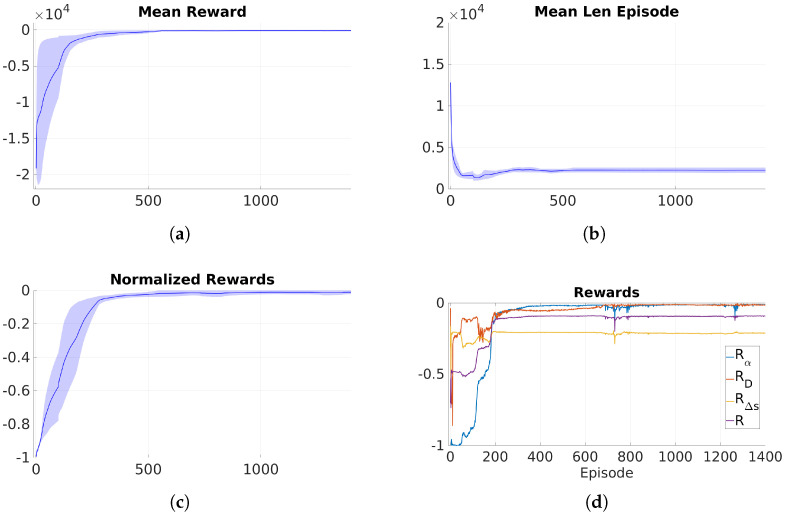
Training metrics: (**a**) mean episodic reward, (**b**) mean episode length, (**c**) normalized reward over the course of training. (**d**) Comparison of overall reward (*R*) and partial rewards ($R_{D},R_{\alpha },R_{\Delta s}$) during training.

During training, metrics such as reward per episode, episode length, and the number of episodes to reach a performance level are used to assess algorithm performance and convergence. While reward per episode indicates overall performance, it may be misleading due to varying episode lengths. Instead, evaluating the normalized reward by episode length offers a more accurate assessment of both measurement accuracy and trajectory efficiency. Figure 9 illustrates this, showing a steady increase in mean episodic reward, changes in episode length, and rising normalized reward, with the RL algorithm stabilizing around episode 500.

The training process was performed on an Ubuntu 20.04 system with an AMD DimgreyCavefish GPU and an AMD Ryzen 7 5800X processor (AMD, Santa Clara, CA, USA). Training for the 1400 episodes took approximately 56 h.

Figure 9d illustrates the partial and total rewards over time. The figure presents the results of three training sessions using the same parameters. The mean and deviation across the three sessions are provided, demonstrating the stability and reproducibility of the training and algorithm. The reward for profile separation is slightly lower than the others, likely due to its assigned weight. However, the error remains minimal and within acceptable limits, reflecting a deliberate design choice to prioritize key parameters like orientation and distance while still ensuring adequate accuracy in profile separation.

In this experiment, the weights for each partial reward were set based on their importance: orientation ($R_{\alpha }$) and distance ($R_{D}$) were given higher weights of 0.4 each, while profile separation ($R_{\Delta_{s}}$) received a lower weight of 0.2. Although all parameters matter, deviations in profile separation are less critical within acceptable limits, so the focus was on maintaining proper forward movement and prioritizing orientation and distance. Maximum penalties were applied for backward movement or significant deviations from the desired range.

### 3.2. Car Door

**Figure 10:**
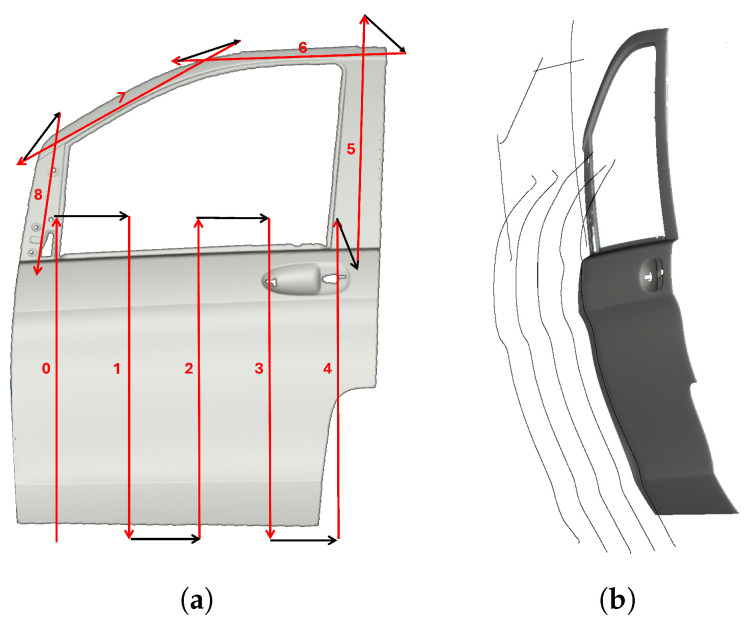
(**a**) Initial boustrophedon-type trajectory for door scanning.inspection passes in red, with numbers indicating the sequence, and transitions in black. (**b**) Resulting trajectories after applying the RL model.

**Figure 11:**
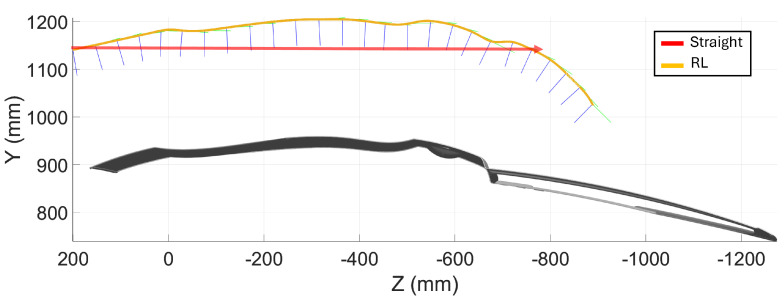
Car door scanning: detail of pass number 3, highlighting specific orientations for the RL trajectory.

The first evaluation piece was a car door (1440 mm × 1060 mm × 190 mm). Figure 10 shows the initial boustrophedon trajectory, with inspection passes in red and transitions in black. The definition of the start and end points of each pass was manually performed using the simulator interface. The reinforcement learning model was applied to optimize the sensor’s orientation and distance during the red passes, ensuring accurate data capture. The white transitions, where no scanning occurs, were not optimized. The optimized trajectories are shown in Figure 11.

**Figure 12:**
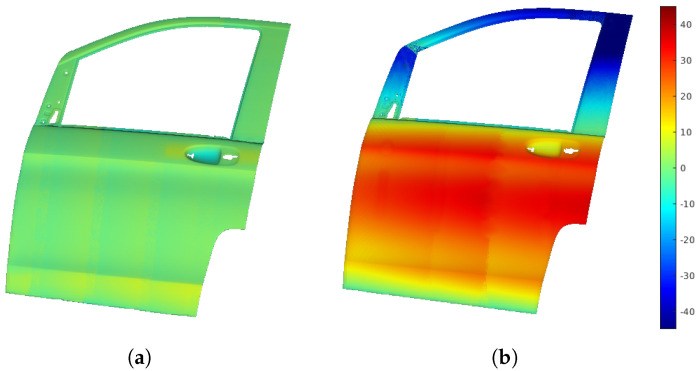
Distance error map, showing the difference between measured and optimal sensor distances. (**a**) Trajectories adapted to the surface by the RL algorithm. (**b**) Straight trajectories defined by start and end points.

Figure 12a displays the point cloud from scanning with the profilometric sensor, showing distance errors as a color map, which reflects the deviation from the sensor’s optimal working distance. Figure 12b shows the distance error map from a traditional boustrophedon trajectory with fixed height and orientation, used for comparison.

**Figure 13:**
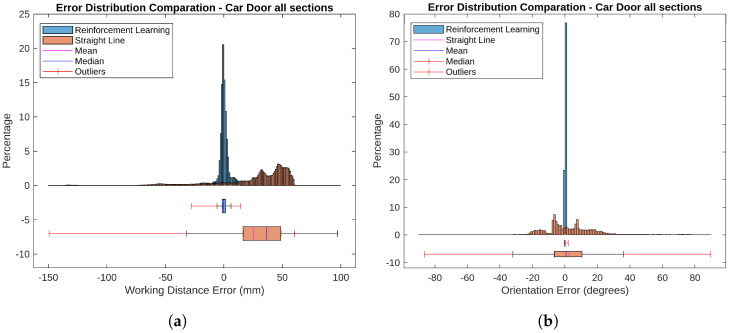
Comparation of the error distribution for all car door sections. (**a**) Distance error. (**b**) Orientation error.

Figure 13 a presents error histograms and boxplots for RL-optimized and straight-line trajectories. The histograms illustrate the distribution of distance errors, while the boxplots highlight the error range and outliers. These metrics are calculated with all the points of the scan, allowing for an evaluation of its performance.

For the RL-optimized paths, the average Mean Absolute Error (MAE) was 2.234 mm, with a median error of −0.080 mm. The boxplots show a narrow error range and minimal variability, indicating consistent performance with errors mostly near zero. In contrast, the straight-line trajectories had a higher average MAE of 37.862 mm and a median error of 7.850 mm. The histograms and boxplots reveal a wider error range and greater variability, reflecting less accuracy and more inconsistency compared to the RL-optimized trajectories.

**Figure 14:**
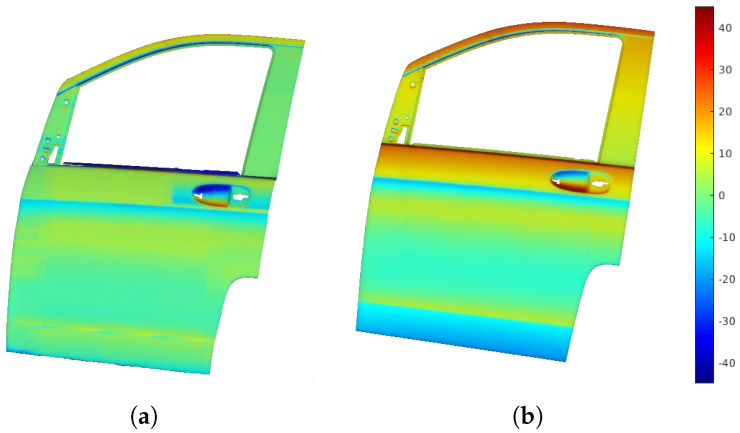
Orientation error maps show angular deviations from the optimal sensor orientation. (**a**) RL-optimized trajectories adapting to the surface. (**b**) Straight trajectories with fixed start and end points.

Figure 14 shows orientation error maps depicting angular deviations from the optimal sensor orientation. Figure 14a illustrates errors for RL-optimized trajectories, while Figure 14b displays errors for straight-line trajectories.

For the RL-optimized paths, the average Mean Absolute Error (MAE) was 2.256°, and the median error was 0.636°. The histograms and boxplots in Figure 13b reveal that most orientation errors were clustered around zero, indicating high precision and consistent performance. In contrast, the straight-line trajectories had a higher average MAE of 13.028°, with a median error of 13.028°. The histograms and boxplots show a wider spread of errors and greater variability, with more pronounced outliers. This reflects less accuracy and greater inconsistency compared to the RL-optimized trajectories.

The RL-optimized trajectories significantly improved the distance measurements and orientation accuracy compared to the straight-line trajectories. The RL approach provided more precise and consistent distance measurements, with lower average errors and reduced variability. It also excelled in handling surface deviations, managing orientation changes more effectively in uneven areas.

**Figure 15:**
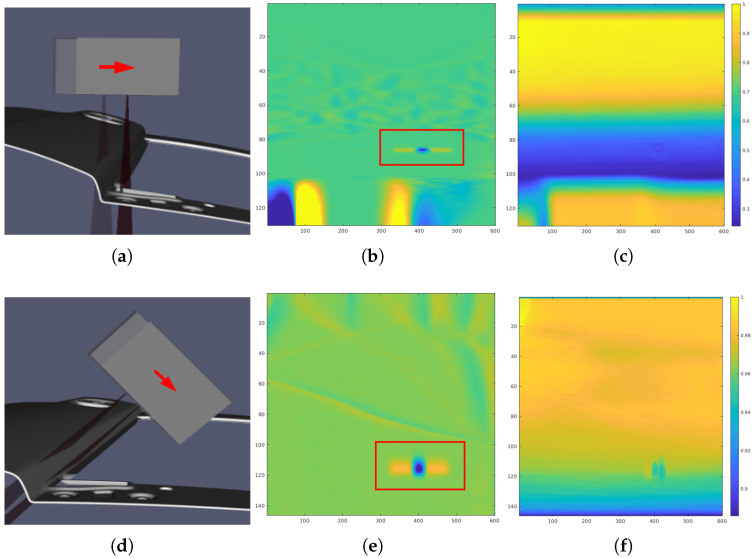
Zoom of a specific area of the scan of the car door. (**a**) Segment of a straight trajectory. Arrow shows movement direction. (**b**) Defect detection results for the straight trajectory. (**c**) Density map from the straight trajectory. (**d**) Segment of an adapted trajectory. (**e**) Defect detection results for the adapted trajectory. (**f**) Density map from the adapted trajectory. Color scale ranges from [0, 1].

Moreover, in Figure 15, a zoomed-in section of a specific area from the global scan is presented. In this region, a bump-type defect was intentionally introduced to analyze how the type of trajectory affects defect detection. This area was chosen because it is small and has a very pronounced orientation. The second column of Figure 15 shows the results of the defect search in the scans, highlighted by a red rectangle. The third column presents normalized density maps in $\mathrm{points}/{\mathrm{mm}}^{2}$, which offer a detailed visualization of the distribution of points in the scanning trajectories.

The simple trajectory demonstrated a low point density in the defect region due to the scanning angle. In contrast, the RL-optimized trajectory achieved a higher point density at the same scanning speed. This increase in point density facilitates a clearer visualization of the defect, significantly enhancing its detection and characterization during the inspection process.

### 3.3. Pen Holder

**Figure 16:**
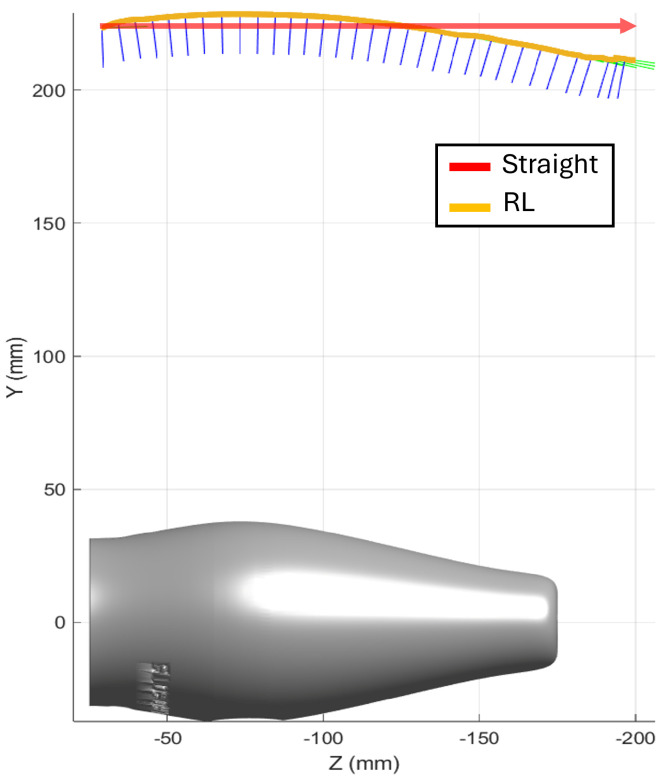
Cad model of the pen holder with both the straight–line scanning trajectory and the RL–optimized trajectory shown.

This subsection details the results from the pen holder scanning experiments. The dimensions of the pen holder are 150 mm × 75 mm × 75 mm. In line with previous sections, a comparison is made between two scanning trajectories: a straight trajectory, moving from a start point to an end point, and a trajectory optimized by the reinforcement learning algorithm, see Figure 16. Additionally, the results from executing these trajectories are presented for both simulation and real-world conditions, utilizing the previously defined inspection system.

#### 3.3.1. Simulation

**Figure 17:**
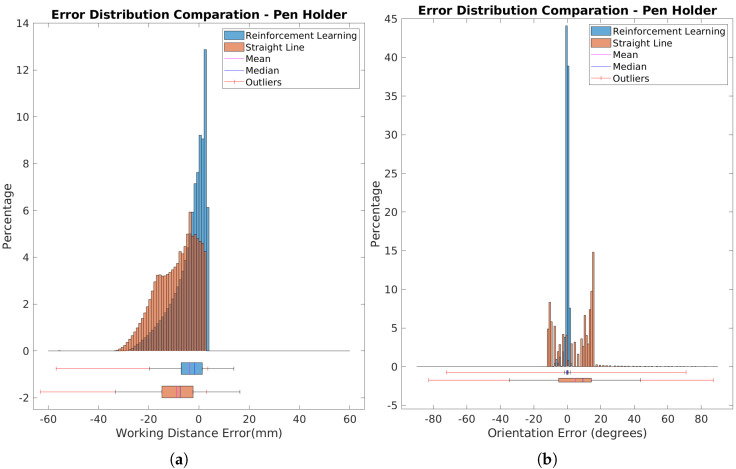
Comparison of error distribution for the pen holder experiment. (**a**) Distance error. (**b**) Orientation error.

Figure 17a shows the distribution of distance errors for the two different trajectories. The RL-optimized trajectory shows significantly smaller errors compared to the straight-line approach. The negative errors, particularly on the lateral areas of the pen holder, are due to its shape of revolution. This type of geometry makes it difficult to achieve zero error across all points, as the sensor cannot perfectly follow the surface contours at every angle. As a result, the goal is to minimize the average distance errors along the scanning path, ensuring overall accuracy.

The distance error comparison reveals that the RL-optimized trajectory performs significantly better than the straight-line approach, with a lower MAE of 2.438 mm versus 6.574 mm. The RL trajectory also shows a smaller median error (2.835 mm compared to 3.415 mm), indicating more consistent and accurate measurements with a narrower error range.

The orientation error comparison based on the boxplot and histogram in Figure 17b demonstrates that the RL-optimized trajectory significantly outperforms the straight-line approach in maintaining accurate sensor alignment. For the RL-generated trajectory, most orientation errors are concentrated around zero, indicating precise and consistent sensor orientation. In contrast, the straight-line trajectory exhibits a much wider spread of errors, ranging from approximately −10° to 20°.

The RL-optimized trajectory achieves a notably lower MAE of 0.707° compared to the straight trajectory’s higher MAE of 11.460°. Similarly, the median error for the RL trajectory is just 0.373°, whereas the straight trajectory has a much larger median error of 10.942°. Although there are some outliers for the RL approach, likely due to specific geometric features such as the hole on the pen holder’s left side, these occurrences are relatively rare.

#### 3.3.2. Real Experiment

**Figure 18:**
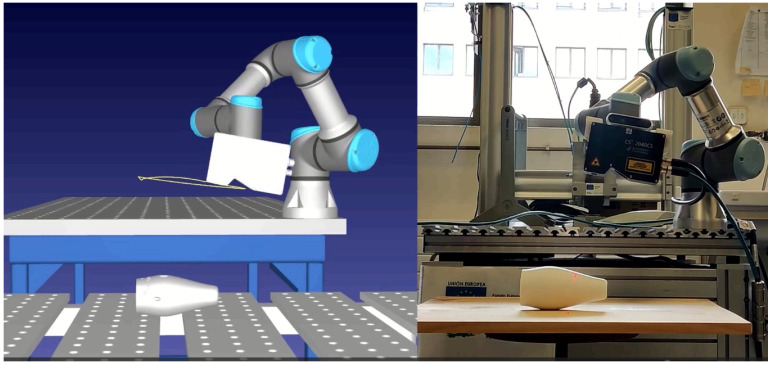
Pen holder scan: simulation vs. reality.

Figure 18 shows a comparison between a real-world experiment and its simulated counterpart, illustrating the robot and sensor scanning a target piece under identical inspection conditions. This highlights the practical alignment of the RL-optimized trajectories with their simulated equivalents.

**Figure 19:**
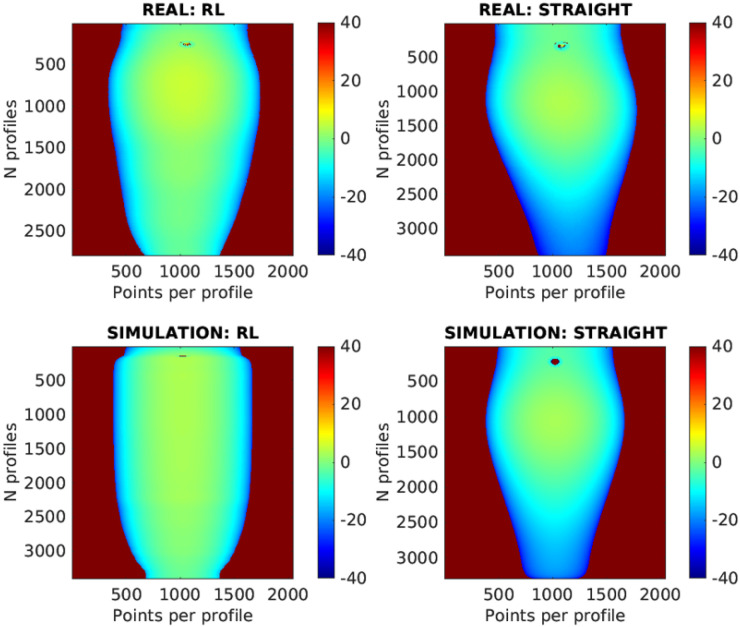
Pen holder scan: 2D images of scanning results comparing RL and straight trajectories in both real and simulated scenarios.

The RL-optimized trajectory is further compared to traditional straight-line paths in Figure 19. The scanning results are displayed as 2D images, with the X-axis representing points per profile, the Y-axis showing the number of profiles, and color gradients indicating deviations from the optimal working distance. The number of profiles differs between the RL and straight trajectories because the straight trajectory scans from a starting point to an endpoint at a constant speed, which in this case was slower than the RL trajectory.

In the images, the results from the simulation and the real-world experiments show a high degree of similarity. This indicates that the simulation effectively replicates the real-world conditions. However, some differences can be observed, which are likely due to the initial calibration of the piece.

In real-world testing, the RL trajectory achieved a Mean Absolute Error (MAE) of 2.422 mm and a median error of 1.798 mm, closely matching the simulated MAE of 2.438 mm and median error of 2.835 mm. For the straight-line trajectory, the real-world MAE was 6.965 mm with a median error of 3.172 mm, compared to the simulated MAE of 6.574 mm and median error of 3.415 mm. These findings underscore the RL model’s effective transfer from simulation to practice, demonstrating superior accuracy and consistency in both simulated and real-world applications.

### 3.4. Parrot Drone

**Figure 20:**
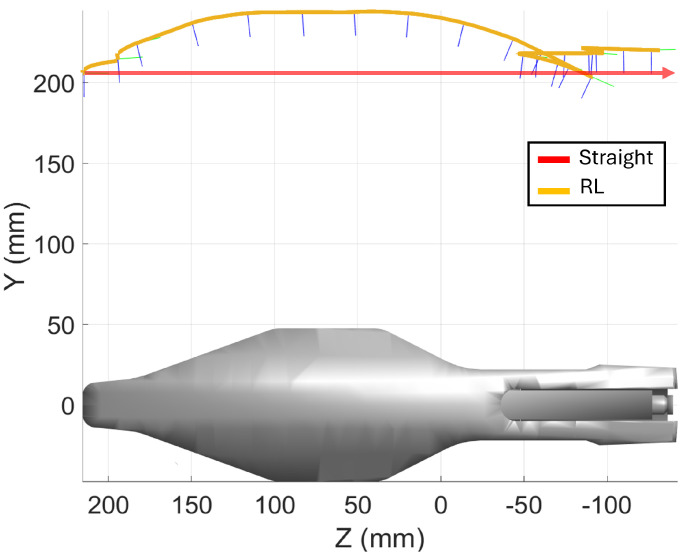
Cad model of the drone with both the straight-line scanning trajectory and the RL-optimized trajectory shown.

The inspection of the Parrot drone, measuring 350 mm × 95 mm × 65 mm, focuses on scanning the flat top surface. Figure 20 displays both trajectories: (a) the straight-line trajectory and (b) the RL-optimized trajectory. The RL-optimized path includes backward movements to adjust the sensor’s orientation, ensuring consistent distance and measurement accuracy throughout the scan.

#### 3.4.1. Simulation

**Figure 21:**
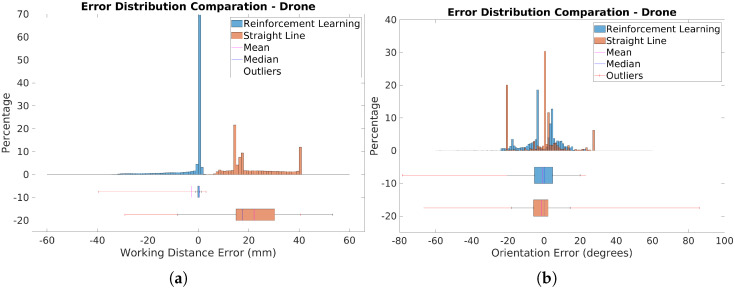
Comparison of error distribution for the drone experiment. (**a**) Distance error. (**b**) Orientation error.

Figure 21a presents histograms and boxplots of distance errors for the RL-optimized and straight-line scanning trajectories. The RL-optimized trajectory shows most errors clustered around very low values, close to zero, indicating high accuracy. Some outliers with higher errors, mainly in the upper part of the drone, are due to its curvature. Despite these outliers, the RL trajectory generally maintains closer proximity to the optimal distance.

In contrast, the straight-line trajectory exhibits a broader range of errors and higher variability. The RL-optimized trajectory achieved a Mean Absolute Error (MAE) of 3.222 mm, significantly lower than the 22.205 mm for the straight-line path. The median error was also substantially better for the RL trajectory, at 0.325 mm compared to 17.426 mm for the straight-line trajectory. This demonstrates that the RL approach provides more consistent and accurate distance measurements.

Figure 21b displays histograms and boxplots comparing orientation errors for the RL-optimized and straight-line trajectories. The RL-optimized trajectory exhibits a more uniform error distribution with fewer extreme values, indicating more consistent sensor alignment. The RL approach notably reduces orientation errors in critical areas, with median errors of 7.269°, compared to 8.977° for the straight-line trajectory.

**Figure 22:**
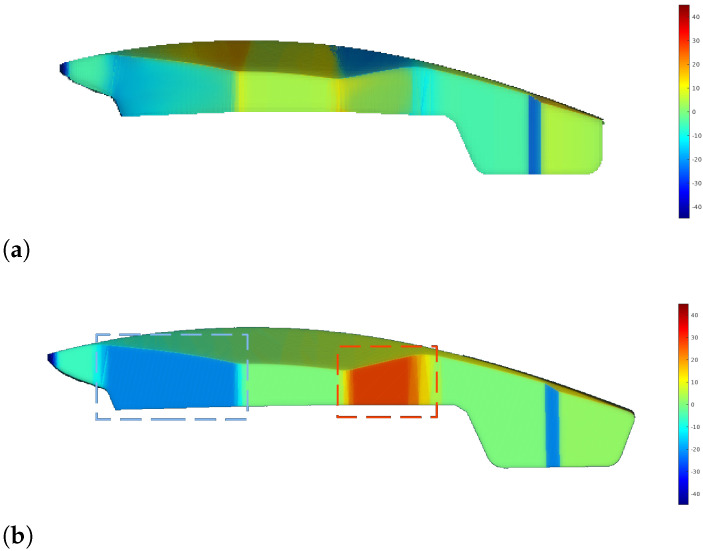
Orientation error map showing deviations from optimal sensor orientation: (**a**) RL-generated trajectory. (**b**) Straight trajectory. Problematic areas are marked in blue and orange.

The straight-line trajectory shows two prominent peaks in the histogram at extreme values of −20° and 27°, reflecting issues in specific regions. In contrast, the RL-optimized trajectory significantly smooths these peaks, demonstrating improved error distribution. The problematic regions are clearly visible in the error map in Figure 22. The RL-optimized trajectory notably reduces errors in these areas. Specifically, in region 1 (blue area), errors decrease from −20° to −8°, and in region 2 (orange area), from 27° to 4°. While overall error metrics are similar, the RL approach significantly improves error reduction and consistency, particularly in critical areas, as evidenced by its lower median error and smoother error distribution.

#### 3.4.2. Real Experiment

**Figure 23:**
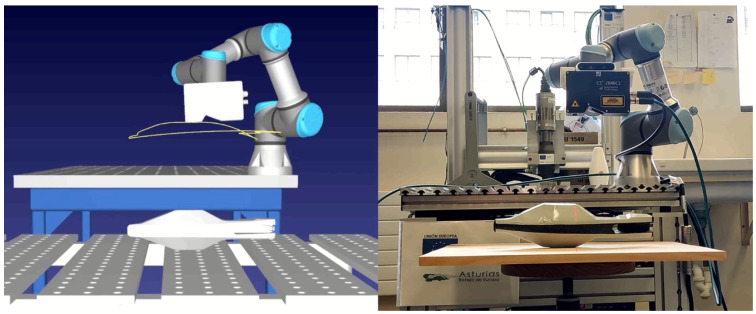
Parrot drone scan: simulation vs. reality.

Figure 23 presents a side-by-side comparison of a real-world experiment and its simulated version, showcasing the robot and sensor as they scan a target piece under consistent inspection parameters. This demonstrates how well the RL-optimized trajectories translate from simulation to practical application.

**Figure 24:**
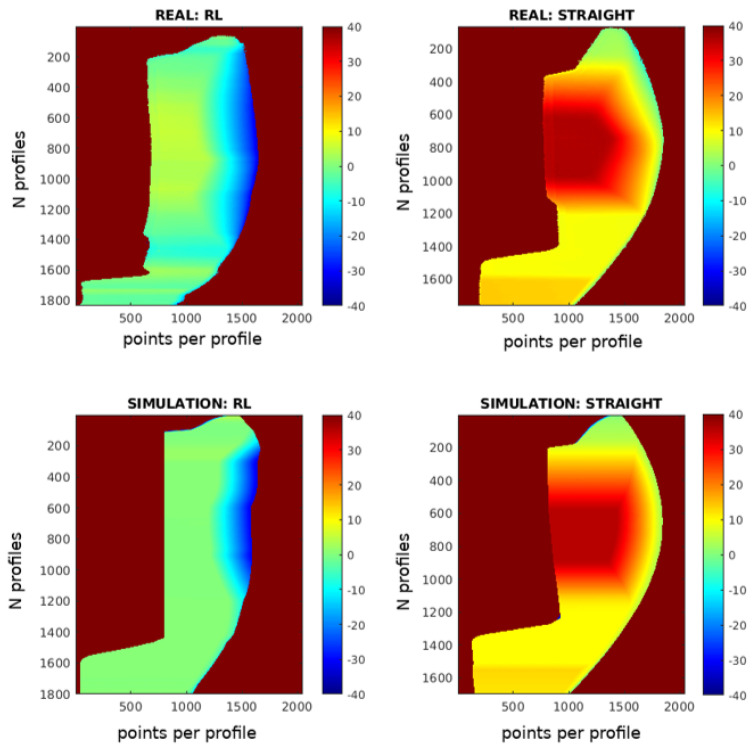
Parrot drone scan: 2D images of scanning results comparing RL and straight trajectories in both real and simulated scenarios.

Figure 24 compares real and simulated scanning results for the RL-optimized and straight-line trajectories. The images reveal a significant similarity between the results from the simulation and the real-world experiments. This suggests that the simulation accurately replicates the real-world conditions.

The results show that the RL trajectory performs similarly in both real and simulated environments, with a real-world MAE of 3.298 mm and a simulated MAE of 3.222 mm. The median error is also close, with real-world values at 2.500 mm and simulated values at 2.600 mm. For the straight-line trajectory, the real-world MAE is 21.570 mm compared to the simulated MAE of 22.205 mm, and the median error is 18.643 mm in the real world versus 17.416 mm in the simulation. This consistency between real and simulated metrics validates the accuracy of the simulation and suggests that the RL method provides reliable performance predictions for real-world applications.

A visual demonstration of the experiments conducted is available in [32].

## 4. Discussion

This paper introduces a method for generating inspection trajectories for laser profilometric sensors using Reinforcement Learning (RL). The approach aims to enhance the scanning process by dynamically adjusting the sensor’s position and orientation to maintain optimal alignment with the inspected surface. A simulated environment, based on our previous work, was used for training. The state space included sensor position and orientation, while additional parameters like profile distance and scan spacing were considered. The action space involves increments in sensor position and tilt, and the reward function focused on sensor distance, alignment, and scan spacing. The RL model was validated through simulations and real-world tests with a UR3e robotic arm, demonstrating its capability to produce accurate, high-quality inspection trajectories and effectively cover surfaces.

In this work, we employ the PPO algorithm due to its demonstrated success in similar applications, particularly in robotic control and view planning for inspections. PPO is known for its balance between implementation simplicity, sampling efficiency, and robustness in constrained environments. However, it is important to note that the choice of PPO is not our main contribution. Instead, our focus lies in optimizing the inspection trajectory through tailored problem formulation and system integration.

For real-world validation, we used smaller pieces due to the UR3e’s limited reach, which restricted us to single linear scanning paths. The car door, which was successfully used in simulations, could not be employed in real-world experiments because its size exceeded the workspace and handling capabilities of the available robotic system. Instead, we selected two representative objects: the Parrot drone case and the pen holder. These parts, despite being smaller, provided sufficient geometric diversity to effectively evaluate the adaptability and robustness of our methodology.

However, our approach is not constrained to small components. In simulations, we successfully applied our reinforcement learning model to larger surfaces, such as the car door, using a boustrophedon scanning pattern—a widely used trajectory for full surface coverage. The RL model dynamically optimized each scanning pass, ensuring that the sensor maintained an optimal position throughout the process. This demonstrates that our method is scalable and can be applied to complex, large-scale industrial components, provided that the robotic system has an adequate workspace.

The scanning trajectories generated by our approach are highly adaptable to any robotic system with sufficient reach, thanks to small, precise increments in position and orientation. The robot’s control software calculates the necessary joint movements based on these incremental commands. Additionally, our method incorporates a penalty mechanism to exclude positions incompatible with the robot’s kinematics, ensuring that only feasible trajectories are generated. This versatility allows the proposed approach to be effectively deployed on various robotic platforms, facilitating high-precision scanning in both small-scale and large-scale inspection tasks.

The dynamic limitation of actions allows adaptation to various applications. In our experiments, backward movement was allowed, though it may not always be feasible depending on the robotic system or part. This flexibility is useful for adjusting the approach to fit various inspection scenarios. It is important to note that while backward movement affects the position of the sensor, it does not impact the position of the profile, which consistently advances.

The proposed reward formulation is based on simple yet effective mathematical models that ensure stable training and meaningful guidance for the agent. Each component of the reward function is carefully designed to reflect key aspects of the inspection process while maintaining computational efficiency. The quadratic nature of the distance and movement rewards provides a smooth and continuous gradient, which enhances learning stability by avoiding abrupt changes in penalties. Additionally, the orientation reward, defined in terms of angular disparity, aligns with established principles in robotic perception. These straightforward formulations not only facilitate implementation but also demonstrate their validity through their direct correlation with the physical constraints of the system.

Additionally, the training process was conducted offline on an Ubuntu 20.04 system with an AMD DimgreyCavefish GPU and an AMD Ryzen 7 5800X processor, requiring approximately 56 h for 1400 episodes. However, this training time is not a critical factor, as it is performed only once. Once trained, the RL model can be applied to different parts without retraining, significantly improving efficiency. Moreover, since the robot executes a precomputed trajectory rather than making online decisions, inference latency is not a limiting factor in our approach and does not impact the inspection process.

In our experiments, we used the same RL model, originally trained on a generic part, to plan the scanning trajectories. This model has demonstrated robustness by performing well on parts with varying characteristics without the need for retraining, highlighting the generalization capability of RL. By training the model on a single reference part, it learned strategies for optimization that can be applied to different geometries, thus enabling the generation of inspection trajectories for pieces with diverse shapes. This flexibility of the RL model makes it more efficient and scalable compared to traditional optimization methods, which often require customization for each specific piece. However, the approach can be refined by retraining the RL model with a part similar to the one being inspected. This would enable more precise trajectory planning and improve accuracy and efficiency in a range of applications.

Inspecting large parts, like car doors, poses a risk of exceeding the sensor’s working range, a challenge especially in automotive manufacturing. Our RL method addresses this by adapting the scanning path to complex geometries, effectively managing errors and ensuring accuracy. Unlike traditional methods, which depend on precise initial alignment and struggle with depth variations, the RL approach dynamically adjusts to the part’s shape, reducing errors and improving inspection reliability.

Figure 15 highlights the importance of focusing on areas with pronounced curvature, where defects such as cracks commonly occur. A zoomed-in section of the global scan shows a bump-type defect introduced in such a high-curvature region. The RL-optimized trajectory achieved not only a higher point density but also improved sensor orientation and distance in these critical areas compared to the simple trajectory. This enhancement in both point density and sensor positioning improves defect visualization and detection, demonstrating the RL-based approach’s effectiveness in identifying issues in defect-prone regions.

A limitation when transitioning from simulation to reality is the need for precise initial alignment of the part to ensure consistent scanning results. While calibration can introduce some variability, it is manageable and aligns with standard industry practices, as parts are typically calibrated relative to sensors and placed in consistent starting positions. Future work will focus on developing methods for aligning the robot more precisely and integrating other 3D sensors to provide approximate geometric information, further improving the robustness of the scanning process.

Reinforcement Learning (RL) offers a valuable approach for advancing automated inspection systems. Future research could focus on improving algorithms to enhance the precision and efficiency of sensor guidance by incorporating techniques like deep learning for real-time trajectory planning and pattern recognition. Refining reward structures, such as including global rewards that balance surface coverage, defect detection, and scanning efficiency, could further improve performance. Additionally, exploring different RL methods, such as policy gradient or actor–critic models, might help identify the most suitable strategies for various inspection scenarios.

Another area for development involves increasing the system’s dimensionality to facilitate the inspection of larger and more complex parts. This could include expanding sensor movement capabilities and adapting trajectory planning to handle multi-dimensional data. Considering alternative trajectory designs, such as spirals or zigzags, may also lead to more effective scanning strategies for specific applications.

## 5. Conclusions

This work presented a Reinforcement Learning (RL)-based approach for generating optimized inspection trajectories using laser profilometric sensors. The proposed method dynamically adjusts the sensor’s position and tilt to maintain optimal scanning conditions, ensuring high-quality data acquisition while adapting to different part geometries.

A simulated environment was used to replicate real-world conditions, where the state space was defined by the sensor’s position, orientation, mean profile distance, direction angle, and scan spacing. The action space consisted of position and tilt adjustments, while the reward function was carefully designed to balance distance, alignment, and scan efficiency.

Experimental results demonstrated the effectiveness of the method in generating efficient, high-coverage inspection trajectories. The RL-trained model generalized successfully to new parts without requiring retraining, highlighting its adaptability and scalability for industrial applications. Real-world validation using a UR3e robotic arm confirmed that the learned policies translate effectively to physical environments.

Furthermore, defining the action space in terms of the sensor’s own coordinate system allows the method to be applied to any robotic system with sufficient reach. Since the trajectory is generated as a sequence of small increments in position and orientation, any industrial robot can execute the movements without requiring specific adjustments to its kinematics.

Compared to conventional approaches, the proposed method enhances adaptability by dynamically optimizing sensor orientation and movement, leading to improved defect detection in critical areas.

## Figures and Tables

**Table 1:** Parameters of the profilometric sensor from its datasheet.

Parameters	Value
Working distance	197 mm
Z range	120 mm
Field of view (X-FOV)	100 mm
Points per profile	2048 pixels
Z resolution	3.0 μm

**Table 2:** Hyperparameters of the PPO algorithm.

Hyperparameters	Value
Neural network architecture	[64, 64]
Activation function	ReLU
Learning rate	0.0003
Update rate	2048 steps
Batch size	64
Discount factor (γ)	0.99
Clip ratio	0.2
Epoch	10

## Data Availability

The data presented in this study are available from the corresponding author on request.
